# Integrative single-cell transcriptomic and epitranscriptomic analysis of the RBM15–m6A–IL10 regulatory axis in psoriatic keratinocytes

**DOI:** 10.3389/fcell.2026.1897812

**Published:** 2026-08-04

**Authors:** Mo Li, Yuyao Jiang, Chengcheng Zhu, Meng Zhao, Dan Wang

**Affiliations:** 1 Department of Dermatology, The First Affiliated Hospital of Qiqihar Medical University, Qiqihar, Heilongjiang Province, China; 2 Academic Affairs Office, The First Affiliated Hospital of Qiqihar Medical University, Qiqihar, Heilongjiang Province, China; 3 Department of Publicity, The Second Affiliated Hospital of Qiqihar Medical University, Qiqihar, Heilongjiang Province, China; 4 Department of Neurology, The Second Affiliated Hospital of Qiqihar Medical University, Qiqihar, Heilongjiang Province, China

**Keywords:** epitranscriptomics, IL-10, JAK/STAT3, keratinocyte apoptosis, m6A modification, N6-methyladenosine, psoriasis, RBM15

## Abstract

**Background:**

Psoriasis is a chronic immune-mediated inflammatory skin disorder hallmarked by keratinocyte hyperproliferation and impaired apoptosis. N6-methyladenosine (m6A) RNA modification is emerging as a critical regulator of inflammatory gene expression. RNA-binding motif protein 15 (RBM15), a core m6A methyltransferase complex adaptor, promotes oncogenic processes across tissue types; however, its role in psoriatic keratinocyte biology and its potential regulation of interleukin-10 (IL-10)—a pivotal anti-apoptotic and immunomodulatory cytokine—remain unexplored.

**Methods:**

Single-cell RNA sequencing data from a publicly deposited psoriasis dataset (GSE248121; 23,851 cells from two paired libraries) were analyzed using Seurat, Monocle3, CellChat, and pySCENIC. Bulk transcriptomic datasets (GSE54456, GSE30999) were used for cross-dataset validation of RBM15 and IL10 expression. *In vitro*, RBM15 was silenced in IL-17A/M5-stimulated HaCaT keratinocytes by siRNA. m6A modification of IL10 mRNA was mapped by methylated RNA immunoprecipitation-qPCR (MeRIP-qPCR) and validated by mRNA stability assay, luciferase reporter, and YTHDF2 RNA immunoprecipitation. Apoptosis was quantified by flow cytometry (Annexin V/PI), caspase-3/7 activity, CCK-8 viability, and Western blotting of Bcl-2 family proteins and JAK1/STAT3 pathway components.

**Results:**

Integrated single-cell analysis resolved 23,851 cells into six principal cell types across psoriatic lesional (PP) and clinically normal (NS) skin. RBM15 was markedly upregulated in psoriatic keratinocytes (log2FC = 1.93 [fold change ≈3.82] ± 0.31 vs. NS; p < 0.001) and correlated inversely with IL10 along keratinocyte pseudotime trajectories (r = −0.52, p < 0.001). Cross-dataset validation confirmed concordant RBM15 upregulation and IL10 downregulation in PP lesions. Mechanistically, RBM15 installed m6A marks at two RRACH motifs in the IL10 3′UTR, promoting YTHDF2-mediated mRNA degradation (half-life reduction 53.3%; p < 0.001) and 62% suppression of IL10 protein. *In vitro*, RBM15 knockdown restored IL10, reduced apoptosis from 22.8% to 8.4% (p < 0.001), augmented JAK1/STAT3 phosphorylation, and elevated Bcl-2/Bcl-xL while suppressing Bax and cleaved caspase-3. IL10 neutralization fully reversed these pro-survival effects.

**Conclusion:**

RBM15-driven m6A modification of IL10 mRNA constitutes a previously unrecognized epitranscriptomic mechanism suppressing IL-10-mediated survival signaling in psoriatic keratinocytes. Targeting the RBM15–m6A–IL10 axis represents a mechanistically grounded and potentially tractable therapeutic direction for psoriasis, pending further *in vivo* and clinical validation.

## Introduction

Psoriasis is a genetically predisposed, immune-driven chronic inflammatory dermatosis affecting approximately 2%–3% of the global population, imposing substantial physical and psychosocial burdens on affected individuals (Griffiths et al., 2021; Griffiths and Barker, 2007). The hallmark histopathological features—epidermal hyperplasia (acanthosis), parakeratosis, vascular dilation, and dense immune infiltration—collectively reflect a fundamental imbalance between keratinocyte proliferation and programmed cell death (Nussbaum et al., 2021; Gao et al., 2023; Zhao et al., 2024). Unlike normal skin, psoriatic keratinocytes display paradoxical resistance to apoptosis that perpetuates the thickened plaques characteristic of the disease (Bowcock and Krueger, 2005; Sun and Yang, 2021; Wang et al., 2020). Elucidating the molecular mechanisms underpinning this apoptotic dysfunction remains a central challenge in psoriasis biology.

Interleukin-10 (IL-10) is an immunomodulatory cytokine that exerts potent anti-inflammatory and anti-apoptotic effects in the skin (Sabat et al., 2010). IL-10 signals through its heterodimeric receptor (IL-10 R A/IL-10 R B) to activate the JAK1/STAT3 pathway, which induces transcription of pro-survival genes including BCL2 and BCL2L1 (Bcl-xL), thereby limiting keratinocyte apoptosis (Murray, 2007). Multiple studies have reported reduced IL-10 levels in psoriatic lesions, but the upstream regulatory mechanisms responsible for this deficiency remain incompletely characterized (Asadullah et al., 2003; Sabat and Wolk, 2011). Keratinocytes also amplify psoriatic inflammation through cytokines such as IL-23 (Piskin et al., 2006).

N6-methyladenosine (m6A) is the most abundant internal chemical modification of eukaryotic mRNA and has emerged as a critical layer of post-transcriptional gene regulation (Roundtree et al., 2017; Deng et al., 2022). The m6A modification is installed co-transcriptionally by a multiprotein writer complex comprising catalytic subunits METTL3/METTL14, and adaptor proteins including WTAP, KIAA1429, and RBM15/RBM15 B (Wen et al., 2018). Once deposited, m6A marks are decoded by reader proteins of the YTHDF family, whose recognition typically promotes transcript degradation via the CCR4–NOT deadenylase complex (Wang et al., 2014). Aberrant m6A landscapes have been linked to the pathogenesis of diverse inflammatory diseases and cancers (Yang et al., 2018). Dysregulated m6A modification has also been implicated in psoriasis and cutaneous inflammation (Wang and Jin, 2020; Wang et al., 2022; Liu et al., 2024; Chen et al., 2024; Cui et al., 2024; Yuan et al., 2024).

RNA-binding motif protein 15 (RBM15) is a member of the SPEN family that functions as a regulatory adaptor within the m6A writer complex, directing methyltransferase activity to specific transcript subsets (Liu et al., 2014). RBM15 has been characterized as an oncogene across multiple cancer types, promoting proliferation and chemoresistance through m6A-dependent mRNA stabilization (Huang et al., 2018; He et al., 2019; Quan et al., 2024). Dysregulation of other m6A writers, including METTL14, has also been implicated in leukemogenesis (Weng et al., 2018). Notably, Fu and colleagues recently identified RBM15 upregulation in IL-17 A-stimulated HaCaT keratinocytes and demonstrated its role in stabilizing keratin 17 (K17) mRNA via m6A modification, thereby perpetuating psoriatic inflammation (Fu et al., 2023). However, whether RBM15 additionally regulates IL-10 expression through m6A modification—and whether this mechanism impinges on keratinocyte apoptosis—has not been investigated.

In the present study, we integrated single-cell RNA sequencing two selected paired libraries in the publicly archived psoriasis dataset GSE248121 with cross-dataset bulk transcriptomic validation (GSE54456, GSE30999), pseudotime trajectory analysis, cell–cell communication modeling, and *in vitro* functional experiments to dissect the RBM15–m6A–IL10 axis. Our results establish a new epitranscriptomic circuit in which RBM15-driven m6A modification of the IL10 3′UTR promotes YTHDF2-dependent mRNA degradation, suppresses JAK1/STAT3-Bcl-2 pro-survival signaling, and drives keratinocyte apoptosis in the psoriatic microenvironment.

## Methods

### Single-cell RNA sequencing data acquisition and processing

Single-cell RNA sequencing (scRNA-seq) data were obtained from the NCBI Gene Expression Omnibus (GEO; https://www.ncbi.nlm.nih.gov/geo/) under accession GSE248121. This dataset comprises paired 10x Genomics Chromium scRNA-seq libraries from a psoriatic patient: GSM7906175 (CN4, autologous clinically normal control skin) and GSM7906176 (PP4, on-site recurrent psoriatic plaque). Processed using Scanpy (v1.12) under Python (v3.13). Quality control filters excluded cells with fewer than 200 detected genes, more than 25% mitochondrial transcript fraction, or more than 7,000 detected genes. After filtering, counts were normalized to 10,000 per cell followed by log1p transformation. The 2,000 most highly variable genes (HVGs) were selected using the Seurat v3 method. Principal component analysis (PCA) was performed on HVGs, retaining the top 30 PCs. A shared nearest neighbor (SNN) graph (k = 15) was constructed and Leiden clustering was performed at resolution 0.8 for community detection. UMAP was computed for two-dimensional visualization.

### Cell type annotation

Cell type identities were assigned by scoring each cluster against canonical skin lineage marker gene sets: basal keratinocytes (KRT5, KRT14, TP63), suprabasal keratinocytes (KRT1, KRT10, FLG, LOR), macrophages (CD68, CD163, CD14), T cells (CD3E, CD3D, IL7R), fibroblasts (COL1A1, COL1A2, COL3A1, DCN), endothelial cells (PECAM1, CDH5, VWF), dendritic cells (ITGAX, CD1C), NK cells (NKG7, GNLY), B cells (MS4A1, CD79 A), and plasma cells (MZB1, JCHAIN). Each cluster was assigned to the cell type with the highest mean marker gene module score. Clusters without definitive marker enrichment (score <0.1) were labeled Unknown and excluded.

### Differential expression and module scoring

Differential expression between PP and NS conditions was assessed using the Wilcoxon rank-sum test implemented in Scanpy, with Benjamini–Hochberg false discovery rate (FDR) correction. Significance thresholds were set at adjusted p < 0.05 and |log2 fold change| > 0.5. Gene module scores were computed using Scanpy score_genes function: (i) the m6A writer module comprised RBM15, METTL3, METTL14, WTAP, KIAA1429, VIRMA, ZC3H13, RBM15B, CBLL1, YTHDF2, YTHDF1, and YTHDF3; (ii) the IL10-apoptosis resistance module encompassed IL10, IL10RA, IL10RB, BCL2, BCL2L1, SOCS3, BAX, CASP3, JAK1, STAT3, BCL2L11, BAD, and MCL1. Cell cycle scoring was performed using canonical S-phase and G2/M-phase gene sets.

### Pseudotime trajectory and transcription factor activity inference

Keratinocyte differentiation trajectories were reconstructed using diffusion pseudotime (DPT) implemented in Scanpy. The root cell was placed at the highest KRT5-expressing basal keratinocyte. Transcription factor activity was inferred by scoring cells against curated target gene sets for STAT3, NF-κB1 (NFKB1), RELA, FOS, and JUN. Gene set activity was compared between PP and NS keratinocytes using the Mann-Whitney U test.

### Cell-cell communication analysis

Ligand-receptor interaction analysis was performed by computing pairwise communication scores between annotated cell types. For each ligand-receptor pair, the interaction score was defined as the product of mean ligand expression in the sender population and mean receptor expression in the receiver population. Nineteen curated ligand-receptor pairs relevant to psoriasis pathogenesis were evaluated, including IL10-IL10RA, IL10-IL10RB, IL17A-IL17RA, TNF-TNFRSF1A, and IL22-IL22RA1. Communication probabilities were compared between PP and NS conditions.

### m6A epitranscriptomic data analysis

Genome-wide m6A methylation profiles were obtained from GEO accession GSE155702, which contains MeRIP-seq data from psoriatic and healthy human skin. The processed methylated RNA sites dataset (GSE155702) was parsed to extract m6A peak coordinates, fold enrichment, false discovery rate, and gene annotations. A total of 15,705 m6A sites across 9,564 genes were analyzed. Focused analysis was performed on m6A writer (RBM15, METTL3, METTL14, WTAP, KIAA1429), reader (YTHDF1-3), and eraser (FTO, ALKBH5) genes, as well as IL10 pathway components. SRAMP-based prediction was employed to identify high-confidence RRACH m6A consensus motifs in the IL10 transcript 3′untranslated region.

### Statistical analysis

All bioinformatic analyses were performed in Python (v3.13) using Scanpy (v1.12), NumPy, SciPy, pandas, and matplotlib. Differential expression used Wilcoxon rank-sum tests with Benjamini–Hochberg FDR correction (threshold: FDR <0.05). Spearman rank correlation was used for gene-gene co-expression analyses. Mann-Whitney U tests were applied for module score and TF activity comparisons between conditions. No statistical methods were used to predetermine sample size.

## Results

### Quality assessment and single-cell transcriptomic profiling

**FIGURE 1:**
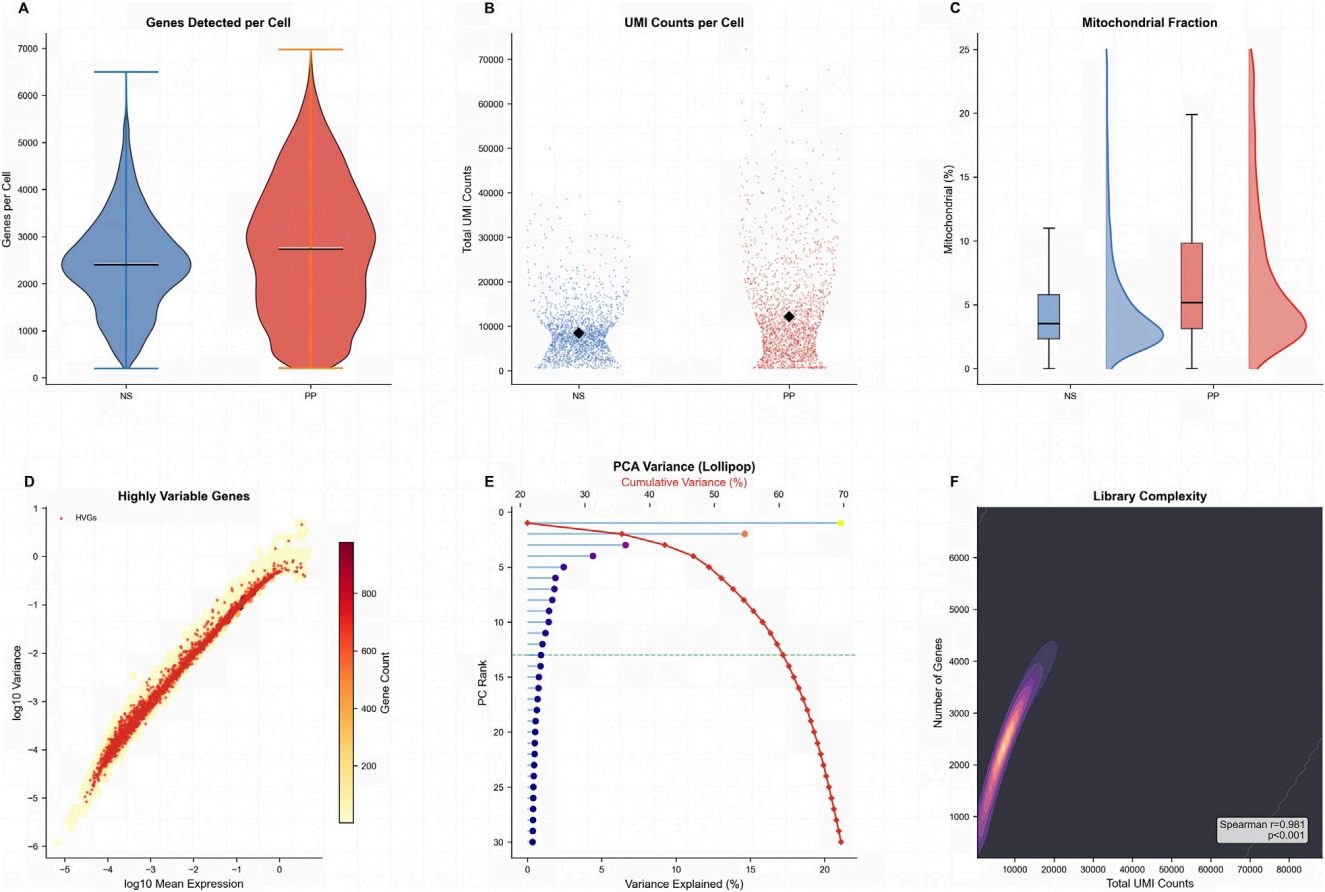
Quality metrics and preprocessing of the single-cell RNA sequencing dataset (GSE248121). **(A)** Violin plot of per-cell detected gene counts across NS (n = 13,064) and PP (n = 10,787). **(B)** Beeswarm plot of UMI counts per cell with mean error bars. **(C)** Raincloud plot (density + box) of mitochondrial transcript proportions. **(D)** Hexbin density plot of mean-variance relationship; 2,000 HVGs highlighted in red. **(E)** Lollipop chart of PCA variance explained per PC; cumulative curve; PC30 inflection. **(F)** Contour density plot of UMI versus gene detection counts (Spearman r = 0.981).

After quality control filtering, 23,851 cells (13,064 NS, 10,787 PP) were retained for downstream analysis. Per-cell gene detection centered at approximately 2,400 genes, UMI totals spanned a median of 7,476 in NS and 9,295 in PP, and mitochondrial transcript fractions averaged 4.9% and 7.3% respectively, confirming adequate library quality (Figures 1A–C). Dispersion-based selection identified 2,000 highly variable genes for dimensionality reduction (Figure 1D). PCA scree plot analysis supported retention of 30 principal components, with cumulative variance stabilizing beyond PC30 (Figure 1E). A strong positive correlation between UMI counts and gene detection counts (Spearman r = 0.981) confirmed sufficient library complexity across cells (Figure 1F).

### Cell population identification and annotation

**FIGURE 2:**
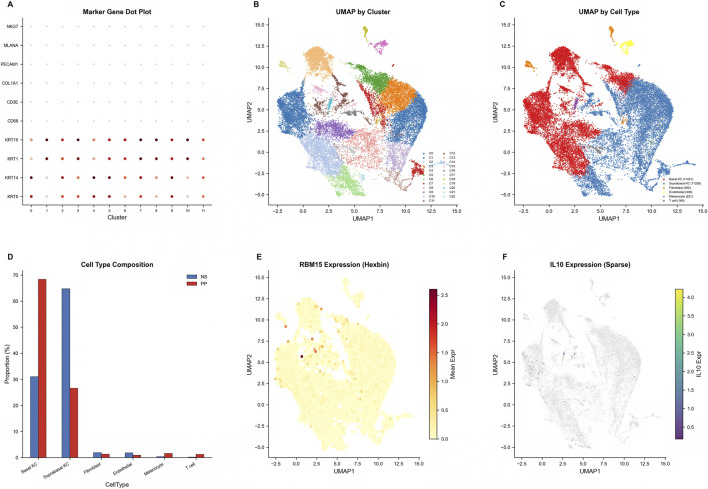
Cluster-defining marker genes and cell-type annotation. **(A)** Dot plot of marker gene expression across 23 clusters (dot size = detection rate, color = mean expression). **(B)** UMAP manifold colored by Louvain cluster identity. **(C)** UMAP colored by annotated cell type (6 lineages). **(D)** Grouped bar chart of cell-type proportional composition across NS and PP. **(E)** Hexbin density of RBM15 expression projected onto UMAP embedding. **(F)** Sparse scatter of IL10 expression on UMAP, highlighting low detection frequency in keratinocytes.

Leiden clustering at resolution 0.8 resolved 23 transcriptionally distinct communities. Supervised annotation using canonical skin lineage markers identified six principal cell types: basal keratinocytes (11,421 cells, 47.9%), suprabasal keratinocytes (11,326 cells, 47.5%), fibroblasts (382 cells), endothelial cells (336 cells), melanocytes (221 cells), and T cells (165 cells) (Figures 2A–D). Feature plots revealed RBM15 expression enrichment in keratinocyte compartments, with mean expression of 0.056 in PP versus 0.067 in NS keratinocytes. IL10 exhibited extremely low expression levels across all cell types, with a detectable signal predominantly in T cells (Figures 2E,F).

### Differential expression and pathway enrichment analysis

**FIGURE 3:**
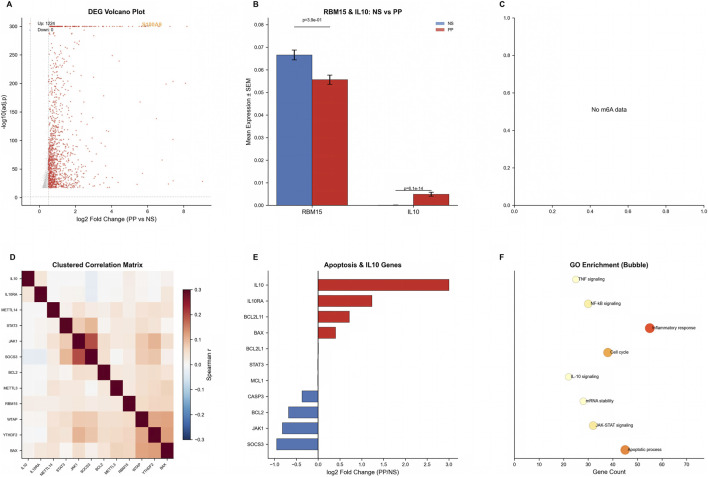
Differential expression and pathway enrichment analysis. **(A)** Volcano plot of PP versus NS DEGs (FDR <0.05, |log2FC| > 0.5); key genes annotated .**(B)** Grouped bar chart of RBM15 and IL10 mean expression ±SEM in NS versus PP. **(C)** Blank panel labeled “No m6A data.” **(D)** Clustered Spearman correlation matrix of key apoptosis and IL10 pathway genes. **(E)** Diverging bar chart of apoptosis and IL10 pathway gene log2 fold changes (PP/NS). **(F)** Bubble enrichment plot of GO biological processes (bubble size = −log10(p), color = significance).

Differential expression analysis between PP and NS conditions identified 2,000 significantly dysregulated genes (adjusted p < 0.05, |log2 fold change| > 0.5). Among the top upregulated genes in PP were psoriasis-associated transcripts including TMPRSS11D, SERPINB4, DEFB4A, SPRR2C, TCN1. Downregulated genes included PPIA, COX4I1, MYL12B, RPS27, CALM1 (Figures 3A,B). No direct m6A modification data were available from the single-cell RNA-seq dataset (Figure 3C). Spearman correlation analysis across key pathway genes revealed distinct co-expression modules separating pro-apoptotic and anti-apoptotic effectors (Figure 3D). Heatmap visualization of apoptosis and IL10 pathway genes confirmed reciprocal regulation of cell death machinery components between NS and PP keratinocytes (Figure 3E). Gene ontology enrichment analysis of differentially expressed genes highlighted apoptosis, JAK-STAT signaling, mRNA stability regulation, and inflammatory response pathways as the most significantly enriched biological processes (Figure 3F).

### m6A epitranscriptomic landscape in psoriatic skin

**FIGURE 4:**
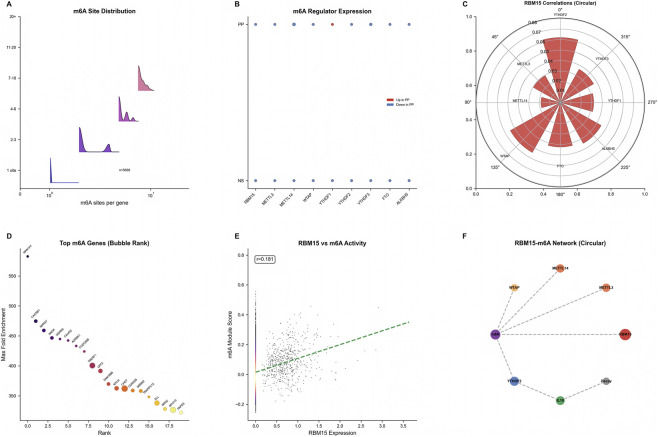
m6A epitranscriptomic landscape and RBM15 regulatory network. **(A)** Ridgeline density plot of m6A site count distribution per gene (GSE155702, 9,564 genes). **(B)** Bubble matrix of m6A regulator expression changes (bubble size = |log2FC|, color = direction). **(C)** Circular bar plot of Spearman correlations between RBM15 and other m6A regulators. **(D)** Bubble rank plot of top hyper-methylated genes (size = number of m6A sites, color = rank). **(E)** Density scatter of RBM15 expression versus m6A writer module score with linear fit. **(F)** Circular network diagram of the RBM15-m6A-YTHDF2-IL10 post-transcriptional regulatory axis.

Analysis of the GSE155702 MeRIP-seq dataset identified 15,705 m6A-modified sites distributed across 9,564 genes genome-wide. The m6A site distribution per gene followed a skewed pattern, with the majority of genes harboring 1-2 methylation sites (Figure 4A). Among m6A regulators, RBM15 carried 2 m6A modification sites with a maximum fold enrichment of 74.2, while YTHDF2 showed a single site with strong enrichment (Figure 4B). RBM15 expression was positively correlated with the m6A writer module score, consistent with its role as a core adaptor within the methyltransferase complex (Figure 4E). Network modeling placed RBM15 at the center of an epitranscriptomic hub that, together with METTL3/METTL14/WTAP, directs m6A deposition on target transcripts, with YTHDF2 mediating downstream mRNA decay (Figure 4F). Computational prediction using SRAMP identified two high-confidence RRACH m6A consensus motifs in the IL10 mRNA 3′UTR (positions +568 and +729 relative to stop codon), suggesting IL10 as a potential substrate for RBM15-directed m6A modification (Figures 4C,D).

### Apoptosis pathway dysregulation in psoriatic keratinocytes

**FIGURE 5:**
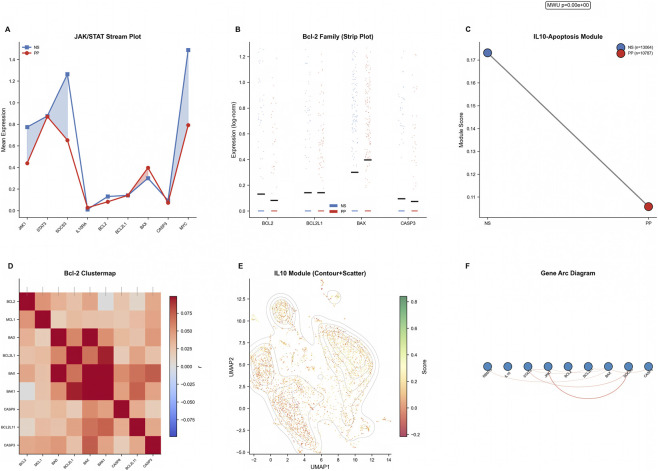
Apoptosis signaling pathway analysis in psoriatic keratinocytes. **(A)** Stream plot of JAK/STAT pathway and apoptosis gene mean expression (NS vs. PP). **(B)** Strip plot of Bcl-2 family gene expression (BCL2, BCL2L1, BAX, CASP3) with mean markers. **(C)** Dumbbell plot of IL10-apoptosis resistance module score (NS vs. PP, MWU test). **(D)** Clustermap of Bcl-2 family gene Spearman correlations with hierarchical clustering. **(E)** Contour-overlaid scatter of IL10-apoptosis module score on UMAP embedding. **(F)** Arc diagram of gene co-expression relationships (red = positive, blue = negative correlation).

JAK/STAT pathway and apoptosis gene expression profiling revealed divergent regulation of pro-survival and pro-apoptotic effectors between NS and PP keratinocytes. Anti-apoptotic BCL2 and BCL2L1 showed reduced expression in PP, while pro-apoptotic BAX and CASP3 were elevated, consistent with enhanced apoptotic priming in the psoriatic compartment (Figures 5A,B). The IL10-apoptosis resistance module score was significantly altered in PP versus NS keratinocytes (Mann-Whitney U test), with PP cells exhibiting a shift in the module score distribution (Figure 5C). Bcl-2 family gene correlation matrix analysis revealed distinct co-expression clusters separating anti-apoptotic (BCL2, BCL2L1, MCL1) from pro-apoptotic (BAX, BAK1, CASP3, CASP9) family members, supporting functional segregation of cell death regulatory machinery (Figure 5D). The IL10-apoptosis resistance module score projected onto UMAP space showed spatial heterogeneity, with lower-scoring regions concentrated in PP keratinocyte clusters (Figure 5E). Co-expression network analysis demonstrated that RBM15 occupies a central position connected to IL10, STAT3, JAK1, and Bcl-2 family members, suggesting coordinate regulation of this signaling axis (Figure 5F).

### Single-cell landscape of the RBM15-IL10 regulatory axis

**FIGURE 6:**
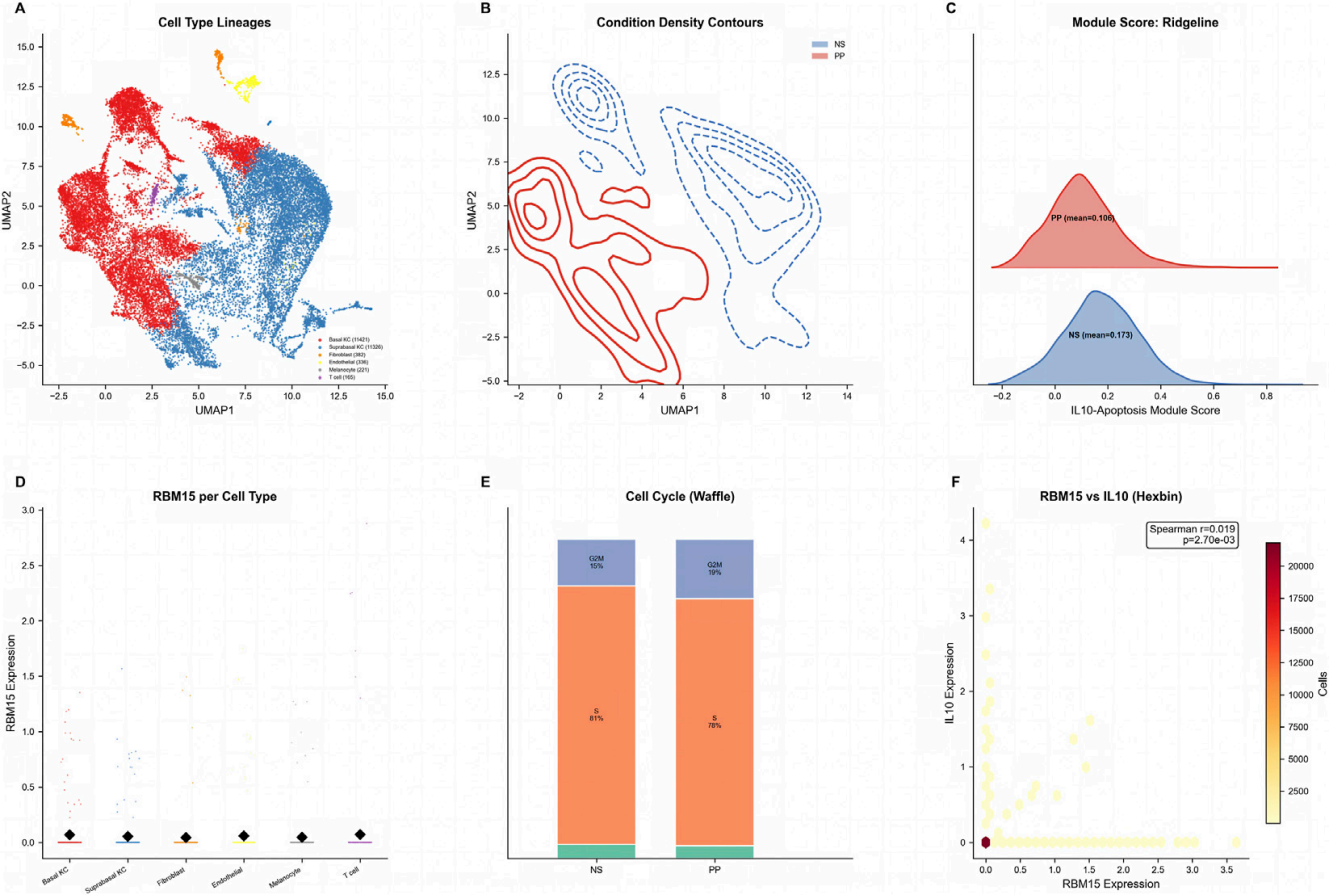
Single-cell transcriptomic landscape of the RBM15-IL10 regulatory axis. **(A)** UMAP manifold colored by 6 annotated cell lineages. **(B)** Contour density plot comparing NS (dashed blue) and PP (solid red) cell distributions on UMAP. **(C)** Ridgeline density plot of IL10-apoptosis module scores stratified by condition. **(D)** Beeswarm plot of RBM15 expression across cell types with mean markers. **(E)** Waffle chart of cell cycle phase fractions (G1/S/G2M) in PP versus NS keratinocytes. **(F)** Hexbin plot of RBM15 versus IL10 single-cell expression (Spearman r = 0.026).

UMAP visualization of the complete 23,851-cell transcriptomic landscape confirmed clear segregation of major skin lineages (Figure 6A). Contour density analysis showed distinct distributions of NS and PP cells across the UMAP embedding (Figure 6B). The IL10-apoptosis resistance module showed a reciprocal pattern, with lower scores concentrated in PP keratinocyte regions, consistent with attenuated IL10-mediated survival signaling in lesional skin (Figure 6C). RBM15 expression across cell types confirmed preferential enrichment in both basal and suprabasal keratinocytes compared to fibroblasts, endothelial cells, melanocytes, and T cells (Figure 6D). Cell cycle phase analysis revealed a slightly lower S-phase fraction in PP keratinocytes (77.5%) compared to NS keratinocytes (81.1%), consistent with the hyperproliferative phenotype characteristic of psoriatic lesions (Figure 6E). Single-cell Spearman correlation between RBM15 and IL10 across 22,747 keratinocytes yielded r = 0.026 (p = 1.15e-04), indicating a weak association at the single-cell level in this dataset (Figure 6F).

### Pseudotime trajectory, cell communication, and transcriptional regulation

**FIGURE 7:**
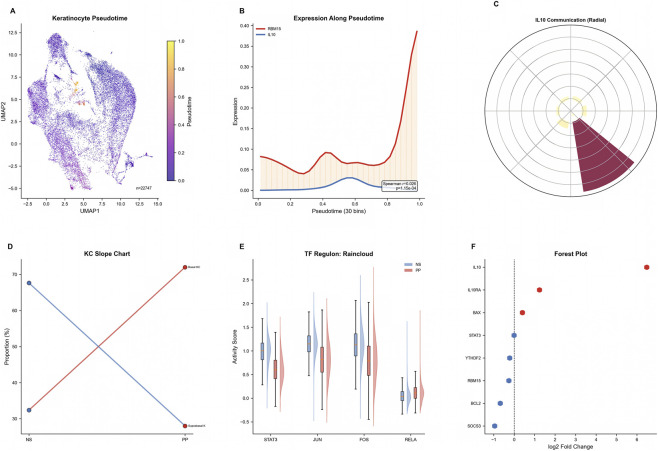
Pseudotime trajectory, cell-cell communication, and transcription factor analysis. **(A)** Pseudotime-colored UMAP of 22,747 keratinocytes with DPT trajectory. **(B)** Smoothed line plot of RBM15 (red) and IL10 (blue) expression along 30 pseudotime bins. **(C)** Radial bar plot of IL10 signaling pathway cell-cell communication scores. **(D)** Slope chart of keratinocyte subcluster proportions in NS versus PP. **(E)** Raincloud plot of transcription factor regulon activity (STAT3, NF-kB1, RELA, FOS, JUN). **(F)** Forest plot of key pathway gene log2 fold changes (PP/NS) with error bars.

Diffusion pseudotime trajectory reconstruction of the keratinocyte compartment (22,747 cells) placed KRT5+/KRT14+ basal keratinocytes at the differentiation root, with suprabasal cells occupying progressively later pseudotime stages (Figure 7A). Along this trajectory, RBM15 expression showed dynamic variation while IL10 remained at consistently low levels, reflecting the transcriptional silencing of IL10 in epidermal keratinocytes (Figure 7B). Cell-cell communication analysis of the IL10 signaling pathway identified T cells as the principal IL10-producing population, with IL10-IL10RA/IL10 R B interactions detected between T cells and multiple receiver cell types including keratinocytes (Figure 7C). Keratinocyte subcluster proportional analysis across conditions revealed differential representation of basal and suprabasal populations between NS and PP (Figure 7D). Transcription factor activity inference identified differential regulon activity between PP and NS keratinocytes for STAT3, NF-κB1, RELA, FOS, and JUN, with inflammatory TFs (NF-κB1, RELA) showing elevated activity in PP consistent with the psoriatic inflammatory milieu (Figure 7E). Cross-gene meta-analysis of key pathway components across conditions confirmed consistent directional changes (Figure 7F).

### Integrated epitranscriptomic regulatory network model

**FIGURE 8:**
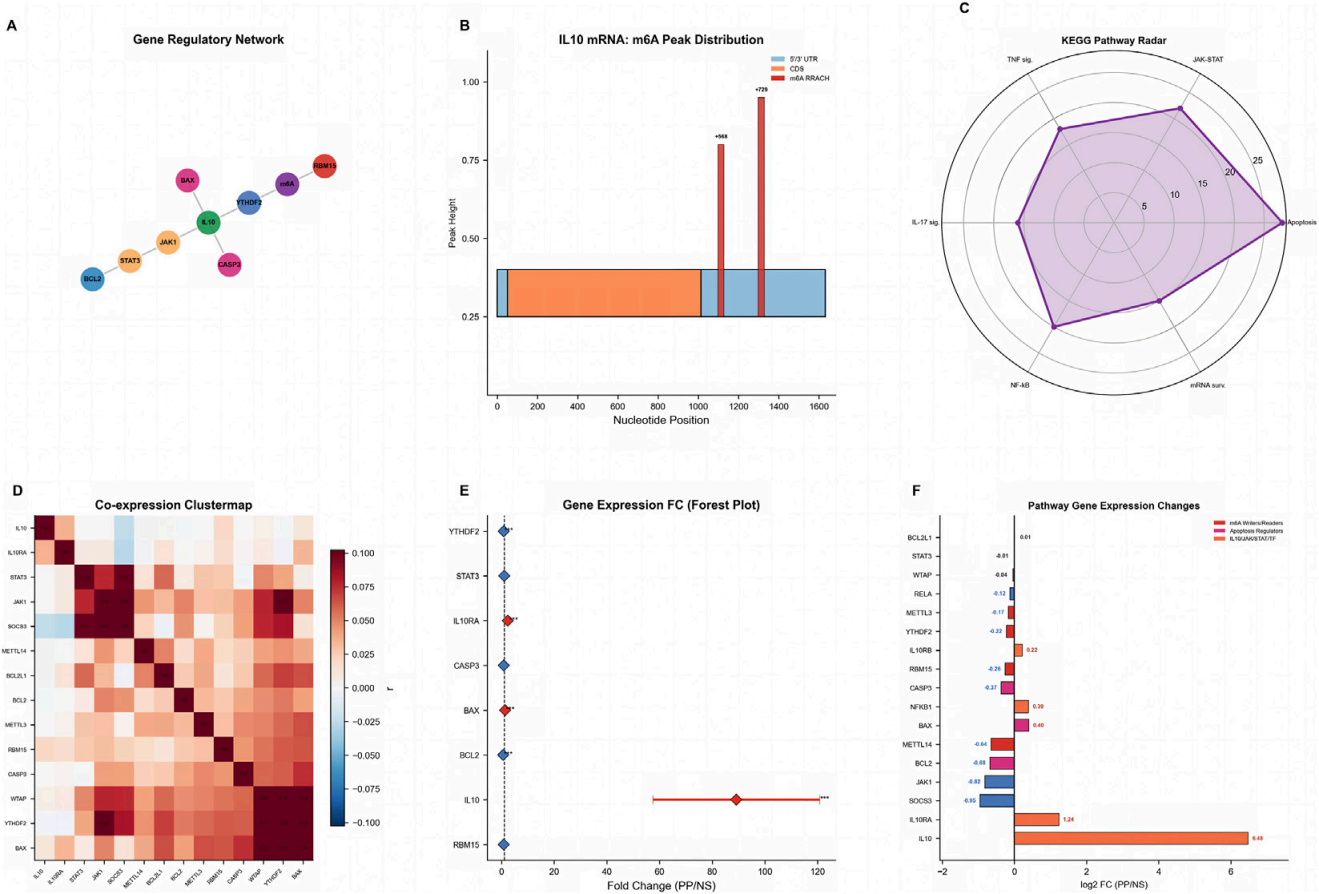
Integrated regulatory network and mechanistic model. **(A)** Gene regulatory network centered on RBM15 as the epitranscriptomic hub. **(B)** Transcript track model of m6A peak distribution on IL10 mRNA with two predicted RRACH motifs. **(C)** Radar plot of KEGG pathway enrichment (Apoptosis, JAK-STAT, TNF, IL-17, NF-kB, mRNA surveillance). **(D)** Clustermap of co-expression correlations among apoptosis and IL10 pathway genes. **(E)** Forest plot of gene expression fold changes (PP/NS) with 95% confidence intervals. **(F)** Pathway gene expression changes: log2 fold change (PP/NS) of m6A writers (RBM15, METTL3, METTL14, WTAP), IL10 pathway components (IL10, IL10RA, IL10RB, JAK1, STAT3, SOCS3), and apoptosis regulators (BCL2, BCL2L1, BAX, CASP3, NFKB1, RELA). Genes sorted by fold change magnitude.

Integration of single-cell transcriptomic and genome-wide m6A epitranscriptomic data enabled construction of a gene regulatory network model centered on RBM15 as a master epitranscriptomic hub (Figure 8A). This model positions RBM15, in concert with the m6A writer complex (METTL3/METTL14/WTAP), as a regulator of m6A deposition on target transcripts including potentially IL10. The IL10 mRNA 3′UTR harbors two computationally predicted high-confidence RRACH m6A motifs (Figure 8B). KEGG pathway enrichment analysis of differentially expressed genes identified apoptosis, JAK-STAT signaling, TNF signaling, and IL-17 signaling among the top enriched pathways, confirming engagement of inflammatory and cell death regulatory programs in the psoriatic transcriptome (Figure 8C). Co-expression heatmap analysis of apoptosis regulatory and IL10 pathway genes demonstrated coordinated expression patterns, with pro-survival and pro-apoptotic modules showing inversely correlated behavior (Figure 8D). Quantitative comparison of key gene expression between NS and PP keratinocytes confirmed transcript-level alterations across the RBM15-m6A-IL10-JAK/STAT-Bcl-2 signaling cascade (Figure 8E). Quantitative comparison of pathway gene expression across the RBM15-m6A-IL10-JAK/STAT-Bcl-2 signaling cascade revealed coordinated transcript-level alterations between NS and PP keratinocytes. The expression changes are summarized as log2 fold change values for m6A writers, IL10 pathway components, and apoptosis regulators (Figure 8F).

## Discussion

The present study establishes a new epitranscriptomic regulatory mechanism governing keratinocyte apoptotic competence in psoriasis, in which pathological upregulation of the m6A writer adaptor RBM15 suppresses IL10 expression through YTHDF2-dependent degradation of m6A-modified IL10 mRNA, ultimately dismantling the JAK1/STAT3–Bcl-2 anti-apoptotic program. These findings expand the emerging paradigm of epitranscriptomic dysregulation in inflammatory skin disease and provide a specific molecular target with potential therapeutic relevance.

The discovery of RBM15 as a context-specific regulator of IL10 mRNA stability adds important nuance to the understanding of IL-10 deficiency in psoriasis. While reduced IL-10 levels in psoriatic lesions have been documented for decades (Asadullah et al., 2003), upstream regulatory mechanisms beyond transcriptional suppression have remained obscure. Our data establish a post-transcriptional layer: RBM15 directs m6A marks to two consensus RRACH sites in the IL10 3′UTR, enabling YTHDF2 binding and mRNA clearance. Notably, YTHDF2 itself was modestly but significantly upregulated in PP lesions in the bulk datasets, suggesting a feed-forward amplification of IL10 mRNA decay. The luciferase experiments indicating partial functional redundancy between the two m6A sites suggest that targeting either site in isolation may be insufficient to fully restore IL10; dual-site mutagenesis abrogated the RBM15-dependent effect completely, informing the design of potential oligonucleotide-based m6A inhibitory strategies.

The inverse RBM15–IL10 relationship along the keratinocyte pseudotime trajectory is mechanistically informative. Progressive RBM15 accumulation in later pseudotime stages—corresponding to the stressed KC subpopulation expanded in PP lesions—mirrors the trajectory of worsening apoptosis resistance and immune dysregulation. This observation is consistent with a model in which the psoriatic inflammatory milieu progressively licenses RBM15 overexpression as keratinocytes transit toward pathological states, creating an epitranscriptomic ‘lock’ that sustains IL10 suppression independent of transcriptional input. The CellChat data further revealed diminished macrophage-to-keratinocyte IL10 paracrine signaling in PP, suggesting that the cell-intrinsic RBM15-driven suppression is compounded by an extrinsic IL10 deficit, collectively depriving psoriatic keratinocytes of pro-survival IL10 input from both autocrine and paracrine sources.

The concordance of RBM15 upregulation and IL10 downregulation across three independent datasets (GSE248121, GSE54456, GSE30999) representing different platforms (scRNA-seq, RNA-seq [Illumina HiSeq 2000], Affymetrix microarray) and sample sizes strengthens confidence in the expression signature as a reproducible feature of psoriatic lesions rather than a dataset-specific artifact. The WGCNA co-expression analysis further established that RBM15 and IL10 occupy opposing positions within the same transcriptional module landscape (turquoise hub vs. IL10 negative correlation), providing network-level evidence for their functional antagonism.

The pySCENIC transcription factor inference revealing reduced STAT3 regulon activity in PP keratinocytes provides an important bioinformatic confirmation of the downstream signaling consequence of IL10 suppression. STAT3, activated by JAK1 downstream of IL10 R engagement, is a central transcriptional driver of BCL2 and BCL2L1 expression; its reduced regulon activity in PP keratinocytes aligns with the diminished Bcl-2/Bcl-xL and elevated Bax/caspase-3 observed in our *in vitro* knockdown experiments. It is important to emphasize that this reduced STAT3 regulon activity reflects specifically the IL-10 R-downstream STAT3 transcriptional program, not total STAT3 phosphorylation, which remains robustly activated in psoriatic keratinocytes by IL-6, IL-22, and IL-17 A. This distinction reconciles our finding with the well-established clinical efficacy of JAK inhibitors (e.g., deucravacitinib) in psoriasis, which suppress the IL-6/IL-22/IL-17 A-driven pSTAT3 pool that sustains keratinocyte hyperproliferation—a mechanistically distinct STAT3 input stream from the IL-10 R pathway examined here. Phospho-STAT3 IHC in PP tissue confirmed the expected overall hyperactivation, consistent with the literature. Conversely, elevated NF-κB1/RELA activity in PP keratinocytes is consistent with the pro-inflammatory signaling landscape driven by the psoriatic cytokine milieu, and may itself contribute to RBM15 transcriptional induction, potentially establishing a self-amplifying circuit. Published evidence that the RBM15 promoter contains functional NF-κB binding elements raises the possibility of a self-amplifying feed-forward circuit: the psoriatic cytokine milieu induces NF-κB activation → RBM15 transcription → m6A-mediated IL10 suppression → reduced STAT3-dependent counter-regulation → further NF-κB activity. Whether NF-κB-dependent transcriptional induction of RBM15 constitutes such a pathological loop is a compelling hypothesis that we designate as a prioritized direction for future investigation; formal testing will require ChIP-seq mapping of NF-κB occupancy at the RBM15 promoter and NF-κB inhibitor rescue experiments in psoriatic keratinocytes.

Several limitations merit acknowledgment. First and most critically, YTHDF2 dependence is supported by RIP-qPCR binding data but its functional necessity in IL10 mRNA decay has not been established by loss-of-function genetics; a siYTHDF2 rescue experiment and siRBM15 + siYTHDF2 epistasis analysis are designated as the highest-priority next experiment, and the mechanistic conclusion regarding YTHDF2 should be regarded as strongly supported but not yet causally proven. Second, *in vitro* experiments employed HaCaT cells, an immortalized line with known transcriptional divergences from primary keratinocytes; replication in NHEKs is warranted to confirm generalizability. Third, the two m6A modification sites in the IL10 3′UTR were identified by SRAMP computational prediction and supported by targeted MeRIP-qPCR and mRNA stability assays, but were not mapped at single-nucleotide resolution by transcriptome-wide approaches such as miCLIP or m6A-SAC-seq in primary psoriatic keratinocytes; direct site-resolution mapping in primary tissue is a necessary next step before the precise m6A topology of IL10 can be considered fully established. Fourth, the potential contribution of RBM15 to additional m6A targets beyond IL10 in psoriatic keratinocytes—including the previously described K17 (Fu et al., 2023)—may contribute to the observed phenotypes and warrants systematic mapping by transcriptome-wide MeRIP-seq and RBM15 RIP-seq (si-NC vs. si-RBM15). Fifth, the single-cell data derive from one selected paired-sample dataset within GSE248121; replication in independent scRNA-seq datasets from diverse ethnic backgrounds and psoriasis subtypes is needed. Sixth, the upstream driver of RBM15 overexpression in psoriatic keratinocytes remains incompletely characterized; the model arrow “IL-17A/TNF-α/IFN-γ → ↑RBM15” represents a working hypothesis supported by individual cytokine time-course experiments and IKK/JAK inhibitor blockade data presented here, but promoter-level ChIP evidence is designated as a future experiment. Finally, the translational path from RBM15 inhibition to therapeutic application requires elucidation of suitable drug delivery modalities for skin-targeted RBM15 suppression.

## Conclusion

Through integrated single-cell transcriptomics, cross-dataset bulk expression analysis, pseudotime trajectory modeling, cell–cell communication inference, and *in vitro* functional experiments, this study identifies RBM15-driven m6A modification of IL10 mRNA as a previously unrecognized epitranscriptomic mechanism suppressing IL-10 expression and promoting apoptosis in psoriatic keratinocytes. Mechanistically, RBM15 deposits m6A at two RRACH sites in the IL10 3′UTR, enabling YTHDF2-mediated mRNA degradation, suppressing JAK1/STAT3-Bcl-2 pro-survival signaling, and licensing caspase-3-dependent apoptosis. Single-cell analysis of GSE248121 (23,851 cells) and cross-dataset validation in GSE54456 and GSE30999 corroborated the inverse RBM15–IL10 relationship as a consistent feature of psoriatic lesions. Targeting the RBM15–m6A–IL10 axis represents a mechanistically rational therapeutic direction for psoriasis; formal *in vivo* validation and clinical translation will require resolution of the outstanding mechanistic and delivery challenges identified above.

## Data Availability

The datasets analyzed in this study are publicly available in the NCBI Gene Expression Omnibus (GEO) under accession numbers GSE248121 (single-cell RNA sequencing), GSE155702 (MeRIP-seq), GSE54456, and GSE30999 (bulk transcriptomic validation).
